# Cognitive household labor: gender disparities and consequences for maternal mental health and wellbeing

**DOI:** 10.1007/s00737-024-01490-w

**Published:** 2024-07-01

**Authors:** Elizabeth Aviv, Yael Waizman, Elizabeth Kim, Jasmine Liu, Eve Rodsky, Darby Saxbe

**Affiliations:** 1 Department of Psychology, University of Southern California, 3620 S. McClintock Ave, Los Angeles, CA 90089 USA; 2 Fair Play Institute, Los Angeles, CA USA

**Keywords:** Cognitive labor, Division of household labor, Maternal mental health

## Abstract

**Purpose:**

Although the division of unpaid household labor has been studied as a driver of global gender inequity, the cognitive dimension of household labor—planning, anticipating, and delegating household tasks—has received less empirical investigation. Cognitive household labor represents a form of invisible and often unacknowledged domestic work that has been challenging to measure.

**Methods:**

Within 322 mothers of young children, we assessed the division of both cognitive (“planning”) and physical (“execution”) household labor within 30 common household tasks using a self-report measure.

**Results:**

We found that while mothers did more of the overall domestic labor than their partners, the division of cognitive labor was particularly gendered, such that women’s share of cognitive labor was more disproportionate than physical household labor. We found that cognitive labor was associated with women’s depression, stress, burnout, overall mental health, and relationship functioning.

**Conclusions:**

This study is one of the first to investigate cognitive labor quantitatively, and the first to investigate cognitive and physical dimensions within the same household tasks. Understanding how cognitive labor affects mothers’ mental wellbeing has important implications for both practice and policy.

**Supplementary Information:**

The online version contains supplementary material available at 10.1007/s00737-024-01490-w.

## Background

The division of household labor is an important but often overlooked driver of global gender inequity. Women carry a disproportionate burden of domestic tasks, and this burden may be linked with their health and wellbeing. In particular, the dimension of cognitive household labor—planning tasks, anticipating needs, and delegating responsibilities—tends to fall particularly frequently to women (Daminger 2019). Cognitive household labor, which may constitute part of the “mental load” (Dean et al. 2022) of home management, can be draining, distracting, and psychologically taxing.

Research exploring the impacts of unpaid labor on maternal mental health and wellbeing has proliferated in the wake of COVID−19. Two recent reviews (Ervin et al. 2022; Seedat and Rondon 2021) documented deleterious effects of unpaid work on women. Ervin et al. (2022) found that both childcare and housework were negatively associated with wellbeing, psychological distress, depression, anxiety, malaise, and sleep problems for working women, but not men. Seedat and Rondon (2021) reported that disproportionate shares of unpaid labor contributed to sex differences in depression. Additional studies have linked the division of domestic labor to women’s marital intimacy and work satisfaction (Choi et al. 2020), burnout (Favez et al. 2023), and relationship distress (Waddell et al. 2021).

Unpaid household labor is less likely to be associated with poor mental health outcomes in men (Offer, 2014). Ervin and colleagues (2022) hypothesize that this discrepancy may be due in part to the burden of the mental load, which includes both cognitively demanding tasks and emotional labor (Dean et al. 2022), all of which falls largely on women. Qualitative research on the cognitive dimension of household labor has indicated that while decision-making is more equally shared between sexes (Daminger 2019), women take on a greater share of all other aspects of cognitive labor, including anticipating needs, identifying options for completing tasks, and monitoring outcomes (Daminger 2019), providing task reminders (Ahn et al. 2017), and establishing the minimum standard for task completion (Mederer 1993). This literature indicates that the discrepancy between male and female participation in these household “management” activities is far greater than the gender gap for physical household tasks such as cooking and cleaning (Mederer 1993).

However, there is surprisingly little empirical research on cognitive household labor, and the existing literature is almost entirely qualitative or theoretical (see Reich-Stiebert et al. 2023 for a review). This dearth of quantitative research may be due to the difficulty in measuring cognitive labor. Much of the research on unpaid domestic labor is conducted with time-use and daily diary data (Ervin et al. 2022), which may miss time spent thinking, planning, scheduling, and organizing household tasks or feeling responsible for household members (Dean et al. 2022). In contrast to qualitative studies, which repeatedly reveal that women report substantially more cognitive household labor, gender differences in cognitive labor look insignificant when studied using time-use measures (Lee and Waite 2005; Offer and Schneider 2011). This may be in part because time-based metrics are not well suited to estimating cognitive labor, which may occur concurrently with other tasks, is “boundaryless,” and might “run in the background” while individuals are engaged in other activities. Time-use studies that do seek to quantify “invisible” labor do so indirectly by testing hypotheses about multitasking, fragmented leisure time, or experiences of feeling pressured (reviewed in Daminger 2019).

However, two recent studies have investigated cognitive household labor using more detailed measures. Petts and Carlson (2023) asked participants to rate how they divided housework and childcare tasks that represented cognitively demanding domestic labor (e.g., planning, organizing, managing), and controlled for physical labor, which included routine housework and childcare tasks that are not cognitively demanding. Rather than distinguish between the cognitive and physical dimensions *within* a given household responsibility, they instead tested domestic tasks that were inherently cognitive (e.g., assigning tasks) and controlled for tasks that they deemed physical rather than cognitive. Consistent with qualitative research, the authors found that mothers spent twice as much time in cognitive labor tasks than fathers, and the division of cognitively demanding tasks fell largely to mothers. These differences were associated with increased stress and depression risk for mothers. Ciciolla and Luthar (2021) similarly used a set of 13 cognitively demanding household tasks, and asked participants to report on their division. They found that the more tasks that mothers rated as “mostly me,” the lower their wellbeing and relationship satisfaction.

### Current study

Within mothers of young children, the current study extends the limited research on the mental load of housework by distinguishing between “planning” or cognitive domestic labor and “execution” or physical domestic labor within 30 household tasks using a self-report measure. For example, an individual may indicate that they are wholly responsible for the “planning” aspects of grocery shopping (e.g., taking stock of pantry items, planning meals for the week, and making a grocery list), but they split the “execution” aspects of grocery shopping (e.g., going to the store) with their partner. We then test whether an inequitable division of household labor is associated with parents’ wellbeing.

We expect that women in heterosexual cisgender relationships will report doing more of both the cognitive and domestic labor than their male partner (hypothesis 1.1), and that this gender discrepancy will be greater for cognitive labor (i.e., task execution will be relatively more egalitarian than task planning; hypothesis 1.2). We also expect that women reporting larger shares of domestic labor will also report worse psychological functioning, wellbeing, and couple relationship quality (hypothesis 2). We will test hypothesis 2 with both physical and cognitive household labor.

## Methods

### Participants

Participants were drawn from a national longitudinal study originally launched in spring 2020 to test how the COVID−19 pandemic affected the transition to parenthood. The primary inclusion criterion was that participants or their partners had to be pregnant at the time of the first survey. All study procedures were approved by the university ethics review board, and participants provided informed consent at study entry.

The initial sample included 681 participants, and 387 participants completed the most recent wave of data collection. Most of the present sample (93%, *n* = 360) identified as female, and most (95%) lived with their partner. Of the 360 female-identifying parents, 354 (98%) were the birthing parent, and 350 (97%) were in a different-sex couple. For the current study, we included participants in analyses if they were female, the birthing parent, and in a different-sex cohabiting relationship or marriage (*n* = 322). Sample demographics can be found in Table 1.

**Table 1:** Sample demographics

Characteristic	*N* = 322
Participant Age, Mean (SD)	35.724 (4.284)
Child Age, Mean (SD)	2.892 (0.166)
Relationship Status, n (%)	
Cohabitating	14 (4.3%)
Married/Domestic Partnership	308 (96%)
Race/Ethnicity, n (%)	
White	257 (81%)
Black/African American	13 (4.1%)
Hispanic/Latine	21 (6.6%)
American Indian or Alaska Native	0 (0%)
Asian or Pacific Islander	18 (5.6%)
Multiracial or Other	9 (2.8%)
Decline, state	1 (0.3%)
Highest Level of Education, n (%)	
Did not complete high school	3 (0.9%)
High School Graduate/GED	12 (3.8%)
Some College	19 (6.0%)
Associate’s Degree	16 (5.0%)
Bachelor’s Degree	88 (28%)
Master’s Degree	98 (31%)
Professional/Doctoral Degree	83 (26%)
Virtual vs. In-Person Work, n (%)	
Fully remote	61 (24%)
Between remote and hybrid	28 (11%)
Hybrid	60 (23%)
Between hybrid and in person	27 (10%)
Fully in person	82 (32%)
Prenatal Annual Household Income, n (%)	
<$25,000	8 (2.5%)
$25,000-$50,000	28 (8.9%)
$50,000-$75,000	42 (13%)
$75,000-$100,000	36 (11%)
$100,000-$125,000	46 (15%)
$125,000-$150,000	51 (16%)
>$150,000	105 (33%)

### Procedure

The current study reports on the sixth wave of the longitudinal study, which took place between May 30 and July 31 2023, when mothers were approximately 36 months postpartum. Previous waves of data collection took place during pregnancy, and again at 3, 6, 12, and 24 months postpartum, and included measures assessing mental health, stress, loneliness, child development, and household composition, but not domestic workload. Findings from previous waves are reported in Morris et al., 2022, 2023 and Morris & Saxbe, 2022, 2023. The current analyses were pre-registered (Aviv et al., 2023), and are the first from the longitudinal study to report on domestic labor.

Participants completed a 45–50 min questionnaire battery of demographic and psychosocial questionnaires online through the Qualtrics platform. The battery included measures assessing mental health, stress, loneliness, burnout, child development, household composition, and domestic workload. For the household labor inventory, we adapted the *Fair Play* deck of cards (Rodsky 2020), each of which represents a domestic task.

### Measures

Descriptive statistics of study measures can be found in Table 2. Supplemental Table 1 shows correlations between study measures and demographics.

**Table 2:** Descriptive statistics of study measures

Measure	*N* = 322
Domestic Labor: Overall, Mean (SD)	5.085 (0.716)
Domestic Labor: Cognitive, Mean (SD)	5.354 (0.775)
Domestic Labor: Instrumental, Mean (SD)	4.819 (0.734)
PROMIS Mental Health, Mean (SD)	3.101 (0.927)
BDI, Mean (SD)	11.484 (9.068)
PSS, Mean (SD)	24.545 (7.908)
DAS, Mean (SD)	24.650 (5.096)
CBI, Mean (SD)	48.408 (19.214)

### Demographic and household characteristics

In the first wave of data collection during pregnancy, we collected information on participants’ gender, age, and income. In the current wave of data collection, participants reported their employment status, and the extent to which their employment was virtual, hybrid, or in-person. Participants also reported whether they lived with their child and/or romantic partner, their child’s sex at birth, their child’s age, the number of children living in the home, and whether their romantic partner is the co-parent to their child.

### Psychological functioning

#### Overall mental health

Participants completed the Global Mental Health index from the Patient-Reported Outcomes Measurement Information System (PROMIS; Cella et al. 2010), a question bank that has been widely used and well-validated. The PROMIS Global Mental Health index includes two questions: the first asks participants to rate their mental health and mood, and the second asks them to rate their satisfaction with their social activities and relationships. Both items are rated on a 5-point scale where 1 is “Poor” and 5 is “Excellent.”

#### Perceived stress

Stress was measured using the Perceived Stress Scale (PSS; Cohen et al. 1983), a widely used 14-item index that measures participants’ general subjective stress over the past month. Items are rated on a five-point scale with 0 = “Never” and 4 = “Very often.” The index produces a single scale of perceived stress, with higher scores indicating a greater degree of stress. Prior research has demonstrated acceptable reliability and validity (Lee 2012).

#### Burnout

Burnout was measured using the Personal Burnout scale from the Copenhagen Burnout Inventory (CBI; Kristensen et al. 2005), a 6 items scale that assesses exhaustion and depletion, with items rated on a 5-point scale ranging from “Always” to “Never.” Higher scores indicate more self-reported burnout. Prior research has demonstrated acceptable reliability and validity for the personal burnout subscale (Kristensen et al. 2005).

#### Depression

Depressive symptoms were measured using the Beck Depression Inventory (BDI-II; Beck et al. 1996), a widely used 21-item self-report questionnaire that assesses mental and somatic complaints related to depression, including loss of pleasure and changes to sleep and appetite. Respondents rate items on a 4-point scale, and responses are summed such that higher scores indicate a greater number of depressive symptoms. Extensive research has demonstrated high internal consistency (Whisman et al. 2000) and validity (e.g., Storch et al. 2004).

### Relationship quality

The Dyadic Adjustment Scale–Short Form (DAS−7; Sharpley and Cross 1982) was used to assess romantic relationship quality. The DAS−7 is a 7-item version of the original 32-item version (Spanier 1976) and produces a single scale of relationship quality. Respondents rate six of the seven items on a 6-point scale, indicating the degree of agreement on relationship issues and the frequency of positive relationship behaviors. The final item asks participants to rate their happiness in their relationship on a 7-point scale (0 = “Extremely unhappy” and 6 = “Perfect”). Higher scores on the DAS−7 indicate a greater degree of positive relationship quality. Prior research has demonstrated acceptable reliability and validity (Sabourin et al. 2005).

### Division of household tasks

We measured participants’ share of household labor using a subset of the *Fair Play* “cards” (Rodsky 2020), each of which represents a household or childcare task category. The *Fair Play* card deck includes 100 cards, each representing a category of household or childcare tasks, and was developed for public dissemination. The original author of the cards interviewed more than 500 families to qualitatively pilot and test the set of tasks. The card deck has sold over 85,000 units, of which 44,000 were sold in the last year alone (NPD Circana BookScan n.d.). Thus, given that this measure is already being used by thousands of families to quantify household labor, it merits empirical assessment. Of the 100 cards, we selected 30 that represented common, frequently performed household task categories that were specifically applicable to parents of two- to three-year-old children. We then gave participants examples of discrete tasks that might fall within the category’s umbrella (for example, both interviewing caregivers and paying babysitters would fall into the category of “household helpers”). Supplemental Table 2 lists all included cards and the examples we provided participants. We asked participants to rate how they divide the planning (who decides what needs to be done) and execution (who actually does the task) for each task. Participants then rated the division of planning and execution on two scales, each ranging from 1 to 7 with 1 being ‘All my partner’ and 7 being ‘All me.’ Participants could also select “N/A” for tasks that do not apply to their household (e.g., taking care of pets). We calculated an overall domestic labor score (cognitive + physical), a physical-only score, and a cognitive-only score by taking the mean of item scores. All three mean scores had excellent reliability (Cronbach’s ⍺ = 0.965, 0.932, 0.943 respectively).

### Data analysis plan

We analyzed the data using a one-sample t-test to determine whether the domestic labor mean is significantly different from 4 (equal share). Next, we used a paired-samples t-test to determine whether the cognitive labor mean is significantly different from the physical labor mean. We then fit a series of multiple linear regression models to test the association between participants’ domestic labor scores and mental wellbeing. We assessed work setting (remote vs. in-person), childcare arrangements, education, ethnicity, and income as covariates in all models. The results were substantively unchanged when we controlled for work setting and childcare arrangements, so we left these variables out for parsimony. Finally, because the cognitive and physical labor scores were highly correlated (*r* = .79), which may lead to problems with multicollinearity, we did not covary for both in the same model.

## Results

**Fig. 1:**
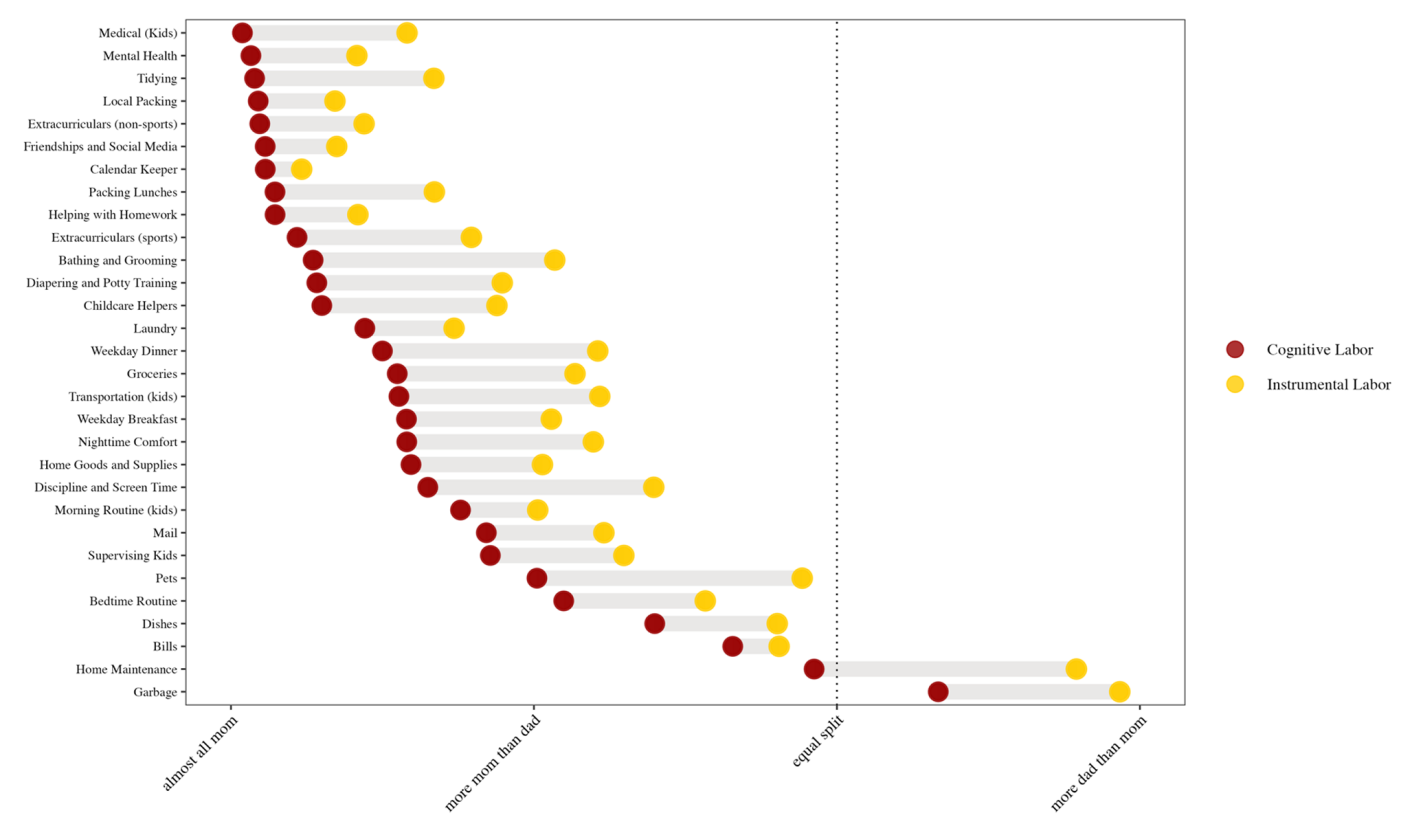
Division of domestic labor by household task

**Fig. 2:**
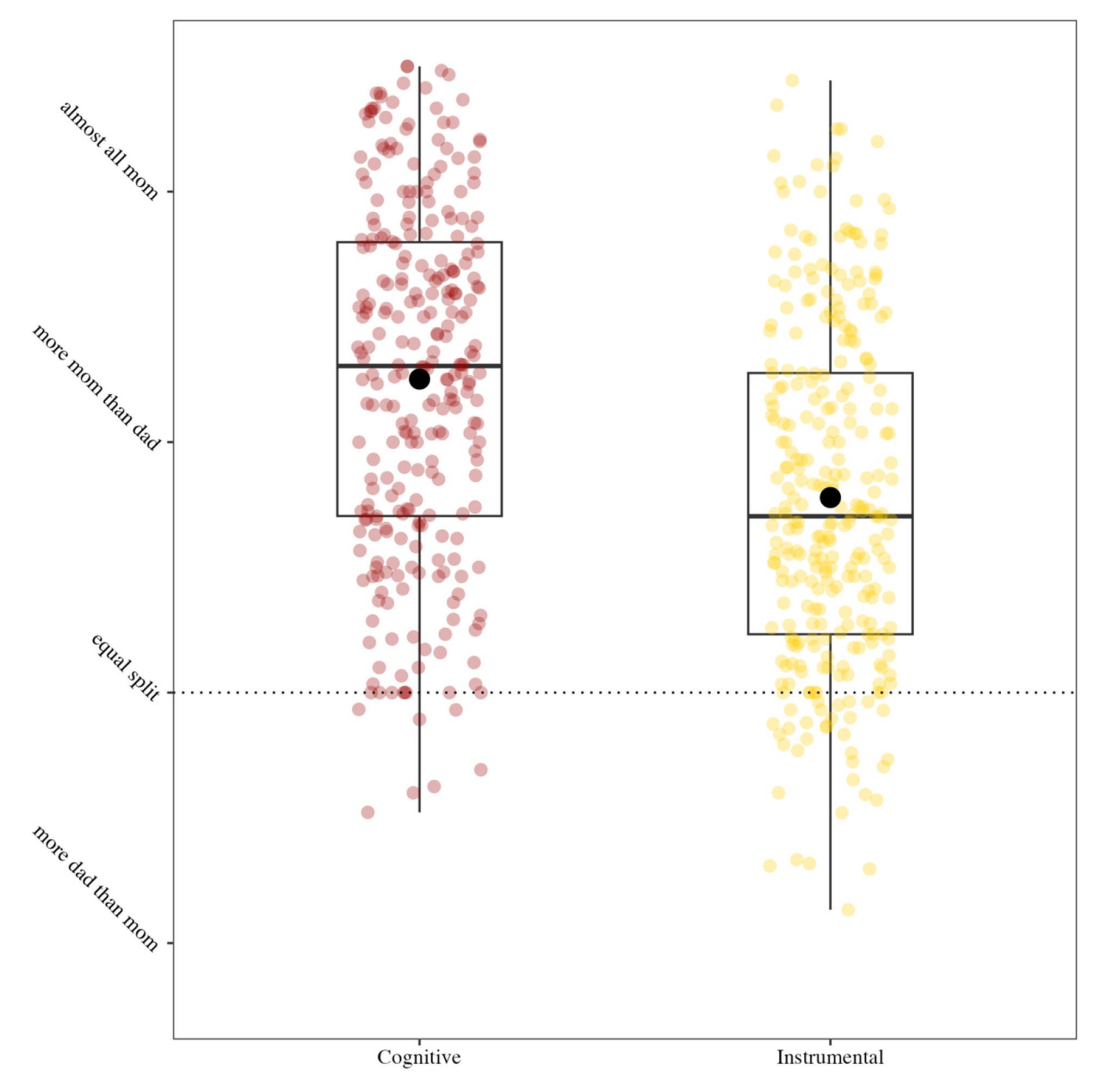
Comparing cognitive and instrumental domestic labor

In support of Hypothesis 1, we found that mothers reported that they are responsible for significantly more of the household burden than their partners (mean = 5.085; t = 26.193, df = 298, *p* < .001; Cohen’s d = 3.03). Overall, mothers reported greater responsibility than their partners for the cognitive labor of 29 out of 30 tasks and the physical household labor for 28 out of 30 tasks (Fig. 1). Taking out the garbage was the only task for which partners were responsible for both the cognitive and physical labor, and home maintenance was the only other task for which partners were responsible for the physical. On average, mothers reported being responsible for 72.57% of all cognitive labor (mean = 5.354) compared to their partners’ 27.43%, and 63.64% of all physical domestic labor (mean = 4.819) compared to their partners’ 36.36%. Consistent with hypothesis 1.2, the difference between mothers’ reported share of cognitive and physical labor is statistically significant (t = 8.67, df = 594.17, *p* < .001; Cohen’s D = 0.71; Fig. 2).

Consistent with Hypothesis 2 (Table 3), we found mothers who reported responsibility for more of the physical domestic labor reported worse relationship functioning (beta =−1.605, *p* < .001). Contrary to our hypotheses, physical household egalitarianism was not associated with depressive symptoms, stress, personal burnout, or overall mental health (Table 3). However, in support of our hypotheses, cognitive labor was significantly associated with all measures of mental health and wellbeing (Table 4). Mothers who reported a greater share of the cognitive workload at home reported increased depressive symptoms (beta = 1.318, *p* = .049), increased stress (beta = 1.765, *p* = .003), increased personal burnout (beta = 4.058, *p* = .005), reduced mental health (beta =−0.142, *p* = .042), and reduced relationship quality (beta =−1.843, *p* < .001).

**Table 3:** Regression models for physical household labor

	Depression			Stress			Burnout			Mental Health			Relationship Functioning		
Characteristic	Beta	95% CI^*1*^	*p*-value	Beta	95% CI^*1*^	*p*-value	Beta	95% CI^*1*^	*p*-value	Beta	95% CI^*1*^	*p*-value	Beta	95% CI^*1*^	*p*-value
Physical household labor	0.139	(-1.269, 1.547)	0.846	0.920	(-0.332, 2.172)	0.149	0.943	(-2.128, 4.014)	0.546	− 0.018	(-0.165, 0.129)	0.811	-1.605	(-2.397, − 0.813)	< 0.001
Income	− 0.390	(-1.063, 0.282)	0.254	− 0.467	(-1.059, 0.124)	0.121	0.023	(-1.430, 1.477)	0.975	0.008	(-0.061, 0.078)	0.817	− 0.192	(-0.567, 0.182)	0.314
Ethnicity	− 0.842	(-3.457, 1.773)	0.527	− 0.103	(-2.424, 2.218)	0.930	-1.446	(-7.112, 4.220)	0.616	0.102	(-0.169, 0.374)	0.458	0.488	(-0.972, 1.949)	0.511
Education	-1.036	(-1.937, − 0.136)	0.024	0.117	(-0.685, 0.920)	0.773	− 0.287	(-2.248, 1.674)	0.774	0.027	(-0.067, 0.121)	0.572	0.329	(-0.176, 0.834)	0.201
R^2^	0.047			0.017			0.003			0.005			0.065		
Adjusted R^2^	0.033			0.003			-0.011			-0.009			0.052		

^*1*^CI = Confidence Interval

**Table 4:** Regression models for cognitive household labor

	Depression			Stress			Burnout			Mental Health			Relationship Functioning		
Characteristic	Beta	95% CI^*1*^	*p*-value	Beta	95% CI^*1*^	*p*-value	Beta	95% CI^*1*^	*p*-value	Beta	95% CI^*1*^	*p*-value	Beta	95% CI^*1*^	*p*-value
Cognitive household labor	1.318	(0.007, 2.629)	0.049	1.765	(0.607, 2.923)	0.003	4.057	(1.222, 6.892)	0.005	− 0.142	(-0.278, − 0.005)	0.042	-1.843	(-2.573, -1.113)	0.000
Income	− 0.371	(-1.038, 0.297)	0.276	− 0.431	(-1.015, 0.153)	0.148	0.076	(-1.357, 1.509)	0.917	0.007	(-0.062, 0.076)	0.848	− 0.243	(-0.612, 0.126)	0.195
Ethnicity	− 0.760	(-3.359, 1.838)	0.565	− 0.014	(-2.308, 2.281)	0.991	-1.284	(-6.878, 4.310)	0.652	0.096	(-0.173, 0.365)	0.483	0.465	(-0.975, 1.905)	0.526
Education	-1.058	(-1.943, − 0.172)	0.019	0.041	(-0.744, 0.827)	0.918	− 0.387	(-2.304, 1.529)	0.691	0.029	(-0.063, 0.121)	0.535	0.481	(-0.012, 0.974)	0.056
R^2^	0.059			0.04			0.028			0.019			0.091		
Adjusted R^2^	0.046			0.027			0.015			0.005			0.079		

^*1*^CI = Confidence Interval

## Discussion

Within a sample of 322 mothers of young children, we found that household tasks showed a gendered division of labor, such that mothers reported that they contributed more than their partners to 28 of the 30 tasks we surveyed. Most strikingly, the cognitive dimension of household labor was particularly gendered; women reported doing significantly more cognitive labor, relative to their partners. Moreover, the reported inequitable division of cognitive labor, in particular, was associated with negative consequences for women’s psychological wellbeing. Whereas the reported division of physical household labor was only associated with relationship quality, cognitive labor was associated with relationship quality, depression, stress, burnout, and overall mental health. These findings point to cognitive household labor as a meaningful correlate of psychological functioning in mothers.

Our finding that mothers report responsibility for significantly more household labor than their partners aligns with prior research on gender disparities in unpaid labor (Bianchi et al. 2012). Furthermore, consistent with the theoretical and qualitative literature on cognitive labor (Daminger 2019), we found that the burden of cognitive labor was even more gendered than physical household labor for all thirty of our measured tasks, and there was a significant overall mean difference between reported cognitive and physical labor. Visual inspection of the data suggests that less cognitively demanding tasks that do not relate to childcare (e.g., garbage, home maintenance, and bills) tended to be divided more equally between mothers and their partners, whereas cognitively demanding, child-related tasks (kids’ healthcare, tidying, and packing kids’ backpacks) were most gendered, with mothers shouldering a larger share of these responsibilities.

As expected, a higher burden of reported cognitive household labor was associated with all measured aspects of psychological wellbeing in mothers. This dovetails with both the existing qualitative research on cognitive labor and the small number of quantitative studies on cognitive labor (Petts and Carlson 2023; Ciciolla and Luthar 2021). The particularly deleterious effects of cognitive labor may be due, in part, to its invisibility: while it is easy to see which partner is chopping vegetables for dinner, the labor of planning a weekly rotation of meals may go unrecognized by other family members, or even by oneself (Daminger 2019). In contrast to previous literature, the division of physical labor was not significantly associated with most measures of psychological wellbeing that we tested, with the exception of relationship quality. The lack of statistical significance may be due, in part, to insufficient statistical power, and the directionality of all effect sizes are consistent with previous literature (Ervin et al. 2022; Seedat and Rondon 2021). However, given that most previous research did not distinguish between cognitive and physical labor, it is possible that prior findings linking household labor with mental health outcomes were driven largely by the cognitive dimensions of housework. While both physical and cognitive aspects of household labor can take time away from other activities, cognitive labor also demands mental resources and may interfere with the ability to concentrate on work or leisure pursuits (Kamp Dush et al. 2018). Thus, though both forms of housework may be demanding, unequal cognitive labor may be especially costly to wellbeing as it may drain one’s mental reserves and enjoyment of other activities, and might also be less likely to generate the sense of accomplishment that might come from completing specific physical housework tasks.

### Strengths, limitations, and future directions

This study contributes to the small body of quantitative research on cognitive labor. However, the study is limited by its well-educated, high-income convenience sample. Given associations between socioeconomic status and both unpaid labor (Craig et al. 2016) and maternal wellbeing (Miller and Carlson 2016), we expect that a lower-SES sample might reveal more striking discrepancies in domestic labor, and a greater impact on wellbeing. This study is also limited by its methods for measuring household labor, which has not been empirically validated. However, our approach to measuring the cognitive and physical aspects of household tasks responds to calls by researchers (Ciciolla and Luthar 2021) for more standardized measures of unpaid labor. Future research should incorporate other existing measures of cognitive labor, including those used by Petts and Carlson (2023) and Ciciolla and Luthar (2021) as well as time-use measures, in order to empirically validate the current measure of the division of cognitive labor. Finally, the study design is cross-sectional and relies on self-report only from mothers and not their partners. We plan to extend this work with future waves of data collection to further explore causal associations between household labor and women’s health.

This is a preliminary study that lays the groundwork for a wide range of future research. While the current study focused on the division of cognitive labor, future work should incorporate other aspects of the construct of cognitive labor that have been outlined in the qualitative literature, such as the degree of its invisibility and its time-boundedness (Daminger 2019). We also hope to extend the current study with future waves of data collection that includes both longitudinal data about the parents in our current sample and data from non-parenting couples. These future data waves will allow us to understand not only how the division of cognitive labor impacts parents’ mental health over time, but will also allow us to understand how the division of cognitive labor might change during parenthood. We also hope to collect partner data in future waves, in particular partners’ income and occupational status, to better understand how couples make decisions about their division of cognitive labor.

Despite its limitations, this project is an important early step in quantifying the “invisible” cognitive labor shouldered by women. It is one of the first studies to investigate cognitive labor quantitatively, and the first to investigate both cognitive and physical dimensions of the same household tasks. It uses Likert-scale reporting rather than time-use data, which may be better suited to estimating cognitive labor (Daminger 2019). Understanding how both cognitive household labor affects mothers’ mental wellbeing has important implications for clinicians treating families. Couples therapists and clinicians treating both mothers and fathers should consider introducing education on cognitive labor, particularly during the prenatal period, in order to raise awareness about cognitive labor’s gender inequity and make “invisible labor” more visible. Future research should test clinical interventions that directly target cognitive labor, such as psychoeducation programs or couples coaching. Furthermore, this study adds to the growing literature supporting the value of policy interventions such as paid paternity leave for public health (Cardenas et al. 2021). Workplace or public policies that support more equitable divisions of labor (e.g., policies that encourage father participation in infant care) may have implications for maternal mental health and wellbeing.

## Electronic supplementary material

Below is the link to the electronic supplementary material.

Supplementary Material 1
