# Supplementary material for: Cognitive household labor: gender disparities and consequences for maternal mental health and wellbeing
Source: Arch Womens Ment Health. 2024 Jul 1;28(1):5–14. doi: 10.1007/s00737-024-01490-w (PMC11761833; doi:10.1007/s00737-024-01490-w)
Supplement: Supplementary file 1 — Supplementary Material 1 [file 737_2024_1490_MOESM1_ESM.docx]

| *Supplemental Table 1. Correlations* | | | | | | | | | | | | | | |
| --- | --- | --- | --- | --- | --- | --- | --- | --- | --- | --- | --- | --- | --- | --- |
|  | | 1 | 2 | 3 | 4 | 5 | 6 | 7 | 8 | 9 | 0 | 11 | 12 | 13 |
| 1 | Total Household Labor |  |  |  |  |  |  |  |  |  |  |  |  |  |
| 2 | Cognitive Household Labor | 0.949^***^ |  |  |  |  |  |  |  |  |  |  |  |  |
| 3 | Instrumental Household Labor | 0.944^***^ | 0.794^***^ |  |  |  |  |  |  |  |  |  |  |  |
| 4 | Relationship Functioning | -0.271^***^ | -0.275^***^ | -0.243^***^ |  |  |  |  |  |  |  |  |  |  |
| 5 | Depression | 0.079 | 0.113 | 0.041 | -0.386^***^ |  |  |  |  |  |  |  |  |  |
| 6 | Stress | 0.138^*^ | 0.170^**^ | 0.092 | -0.408^***^ | 0.736^***^ |  |  |  |  |  |  |  |  |
| 7 | Burnout | 0.107 | 0.161^**^ | 0.042 | -0.375^***^ | 0.699^***^ | 0.723^***^ |  |  |  |  |  |  |  |
| 8 | Mental Health | -0.076 | -0.116^*^ | -0.029 | 0.452^***^ | -0.715^***^ | -0.683^***^ | -0.583^***^ |  |  |  |  |  |  |
| 9 | Income | -0.026 | -0.015 | -0.034 | -0.008 | -0.160^**^ | -0.097 | -0.008 | 0.040 |  |  |  |  |  |
| 10 | Education | -0.073 | -0.005 | -0.140^*^ | 0.096 | -0.189^***^ | -0.038 | -0.024 | 0.054 | 0.533^***^ |  |  |  |  |
| 11 | Race/Ethnicity | 0.052 | 0.067 | 0.031 | -0.044 | 0.033 | 0.022 | 0.028 | -0.031 | 0.055 | 0.038 |  |  |  |
| 12 | Virtual vs. In-Person Work | -0.151^*^ | -0.184^**^ | -0.100 | -0.025 | -0.002 | 0.018 | 0.015 | -0.021 | -0.234^***^ | -0.118 | 0.028 |  |  |
| 13 | Daycare | -0.117^*^ | -0.091 | -0.131^*^ | 0.081 | -0.190^***^ | -0.109 | -0.056 | 0.141^*^ | 0.297^***^ | 0.359^***^ | 0.028 | -0.048 |  |
| 14 | Nanny | 0.055 | 0.057 | 0.046 | 0.006 | -0.040 | -0.016 | -0.033 | 0.099 | 0.097 | 0.057 | 0.055 | -0.002 | -0.183^**^ |

| *Supplemental Table 2.* *Included Fair Play Cards* | | |
| --- | --- | --- |
| **Card** | **Planning Tasks** | **Execution Tasks** |
| Childcare helpers (kids) | - Discussing with others what kind of childcare you need - Sourcing if not family (care.com/websites/word of mouth/emails/FB posts/agency) - Transportation concerns: car seats, boosters, etc. - Interviewing (scheduling, conducting, reference checking) - Scheduling days they will be working - Planning terms including payment – hourly vs. salary, on or off the books, determining payment vehicle/employer | - Hiring (negotiating terms/contract) - Ongoing communication and supervision - Making payments - Purchasing/providing meals and snacks for helper - Reimbursing: Gas, meals, and other reimbursements - Termination issues |
| Dishes | - Paying special care to specialty items like non-stick and cast-iron items | - Washing and drying dishes and pots and pans (by hand) and/or loading and unloading dishwasher - Putting dishes away in proper location |
| Garbage | - Applying to the city/municipality for garbage bins - Taking note of garbage day | - Placing/replacing bag in home trash can - Removing full garbage bags Placing full garbage bags in trash shoot/outdoor bins - Labeling bins - Taking bins back out/in, driving to town dump/placing garbage in alley - Checking bins for overflow trash/keeping bins clean |
| Groceries | - Making a master list - Checking refrigerator and pantry for what is low and adding to list | - Shopping (grocery stores and/or online) - Loading and unloading groceries - Placing items in pantry/refrigerator - Throwing away expired items from refrigerator/freezer |
| Home goods & supplies | - Creating an inventory/making a master list (detergent, cleaning supplies, paper products, batteries, office supplies, bathroom supplies / toiletries/ Ziplock bags, Tupperware, lunch boxes) - Checking supplies for what is low | - Shopping - Loading and unloading goods/supplies - ​​​​​​​Placing items in appropriate area |
| Home maintenance | - Making a master list and keeping track of weekly/monthly/annual home tasks that involve maintenance. - Sourcing (if applicable) | *Outsourced:*   - Hiring - Making payments - Communicating and supervising - Placing orders for parts/broken items - Reimbursing to purchase supplies   *DIY:*   - Plumbing: toilet, sink, garbage disposal, water issues (filters, heater, softeners, mineral deposits, inspections) - Electrical: light bulbs, light fixtures, wiring - Interior: HVAC, air ducts, smoke detectors, vents, insulation, cabinets, appliance management, paint, fireplace/chimney, home security, exterminator - Exterior: roof/siding, tiles, gutters, power washing, pool(cleaning, draining, chemicals), exterior walkway cracks and paint, weather proofing, driveway (snow shoveling etc.) |
| Laundry | - Sorting clothes and reviewing clothing instructions | - Spraying stains pre-wash - Washing/drying - Ironing/steaming - Hanging, folding, putting away |
| Mail | - Knowing when the mail comes - Keeping inventory of stamps and envelopes | - Retrieving mail daily - Opening and sorting mail - Mailing outgoing mail - Shipping and picking-up packages at post office |
| Meals (weekday breakfast) | - Menu planning/recipe selection - Making lists of items needed | - Cooking/preparing - Serving - Putting dishes in sink |
| Meals (kids; school lunch) | - Menu and snack planning and purchasing containers - Log into school portal to order school lunch and/or add money to account | - Preparing and packing into container (including filling daily water bottle and providing utensils/ice packs) - Staying apprised of what child is eating/not eating |
| Meals (weekday dinner) | - Menu planning/recipe selection - Making lists of items needed | - Cooking/preparing - Serving - Putting dishes in sink |
| Tidying up, organizing, and donations | - Developing a system for organizing your physical home: closets, kitchen/pantry, bedrooms, playroom, office etc. and digital home - Taking stock of what children are currently playing with and what you can donate/ hand down - Researching options for where items will be donated or family/friends to hand down to | - Clearing/wiping counters - Putting away toys and shoes - Tidying throw blankets and pillows - Safely storing important documents, including birth certificates, passports and Social Security cards - Gathering items to be donated - Dropping off donations - Purchasing storage and organizational containers - Putting together/building storage container |
| Calendar keeper | - Researching how to keep a shared calendar/joint calendar option/app - Pick a shared calendar option - Conversations/coordinating/tracking invitations with other cardholders | - Adding/deleting appointments - Creating kids schedules for helpers |
| Cash & bills | - Set up auto-pay and/or reminders | - Withdrawing cash for the week - Paying all bills and vendor invoices (auto pay/electronic, Venmo, checks, cash) - Balancing checkbook - Doling out children’s allowance |
| Extracurricular (kids; non-sports) | - Determining child's desired activities - Researching all activity options - Surveying child's friends to see if child can participate with friends - Determining practice schedules if applicable - Managing at home practice and preparation and/or arranging for additional support/coac - Arranging for transportation | - Signing up and registering for activities - Purchasing/storing/updating items for activities - Packing necessary items in bag for practice (including water bottle and snacks) - Volunteering when necessary (snack sign-up, work backstage, etc.) |
| Extracurricular (kids; sports) | - Determining child's desired activities - Researching all sports options (school, gyms and/or community leagues) - Surveying child's friends to see if child can play with friends - Determining practice and game schedules - Managing at-home practice and preparation and/or arranging for additional support/coaching - Researching equipment needs - Arranging for transportation to practices and games | - Signing up and registering for the sports lesson/team - Purchasing/storing/laundering/updating necessary uniforms/equipment/cleats - Packing necessary items in bag for practice and games (including uniform, water bottle and snacks) - Volunteering when necessary (snack sign-up, end-of-season party, etc.) |
| Packing/unpacking (kids; local) | *Backpack/day-out bag:*   - Taking inventory of afternoon out bags (water bottle, snacks, sunscreen etc.)   *Overnight bag*:   - Determine what is needed for overnight bag (e.g., bathroom bag, clothing, sleeping needs, medical (e.g., Epi-Pen)) | *Backpack:*   - Backpack packing. including snack, water bottle, lunch box, homework, library books/textbooks/folders, special items - Backpack unpacking including emptying lunch box and placing in sink - Following up on lost items/lost and found   *Day-out bag:*   - Stocking afternoon out bags   *Overnight bag*:   - Overnight bag packing and unpacking |
| Transportation (kids) | - Coordinating and implementing the commute to/from school, extracurricular activities, enrichment, events, playdates, parties - If school bus, signing up for appropriate route - If public transportation, securing bus/subway pass - If carpool, extra considerations: selecting families that live nearby to carpool with (coordinate booster/car seats); determining schedule for your shift and others’ | - Implementing the commute to/from school, extracurricular activities, enrichment, events, playdates, parties |
| Bathing & grooming (kids) | - Scheduling haircuts - Monitoring supply of bath products, detangler, scissors, toothbrushes and floss and notifying “home goods and supplies” cardholder | - Carrying out haircuts - Managing daily showers/baths including wash/brushing hair/brushing teeth - Cutting nails and toenails - Boo-boo care Age-appropriate add-ons (e.g., deodorant, period care, razors, lotion, hair dryer, etc.) - Conducting lice checks when needed |
| Bedtime routine (kids) | - Encouraging quiet/wind-down activity before bed | - Enforcing lights-out policy at particular time - Reading with kids - Bringing kids to the bathroom - Filling bedside water glass - Pre-bed milk or snack routine |
| Diapering & potty training (kids) | - Determining diapering options (disposable brands or cloth) - Taking inventory of diaper bags (wipes, diapers, creams, changing pad, pacifiers, extra change of clothes, loveys, toys, books, etc.) - Researching, consulting with partner regarding training plan - Informing and enlisting teachers and childcare helpers in potty-training plan | - Purchasing appropriately sized diapers/wipes as baby grows - Stocking diaper bags - Overseeing implementation for potty training |
| Friendships & social media (kids) | - Evaluating child’s social situation to see if they are making connections with others by observing and asking questions - Planning and scheduling outings and play dates with friends | - Carrying out outings and play dates with friends - Monitoring and regulating social media accounts |
| Homework, projects, & school supplies (kids) | - Supervising welcome-to-school assignments (e.g., photos of my family) - Assessing child’s daily/weekly school needs - Helping child prioritize what needs to be done based on after-school activity schedule | - Assisting child when needed and providing emotional support when school is challenging - Supporting and purchasing supplies for school projects - Purchasing/arranging clothes for special days (e.g., school photos, performance concerts, field day, color wars, school theme days) - Ordering/shopping for back-to-school supplies |
| Medical & healthy living (kids) | - Researching, interviewing and selecting pediatrician - Scheduling well checks, vaccine appointments, and annual physicals - Ensuring that child has up-to-date vaccines - Keeping track of questions to ask doctor between well visits - Scheduling child’s sick visits - Determining if specialists are necessary (e.g., eye doctor, GI etc.) - Prevention: researching vitamins and sunscreen (for morning routine cardholder) - Encouraging active lifestyle for all family members, especially exercise | - Purchasing and stocking standard pain reliever/allergy medicines for children (and adults whenever possible) - Keeping specialty medicine up to date, including specialty items like EpiPen and asthma medications/nebulizers - Attending, and taking notes during well checks, vaccine appointments, and annual physicals - Attending child’s sick visits - Filling prescriptions, checking medicine side effects and administering medicine - Manage at-home medical issues such as constipation, sleeping issues, allergies, other GI, asthma, ENT, etc. - Purchasing vitamins and sunscreen - Monitoring family members’ consumption of caffeine, alcohol, fried foods, smoking habits, fatty foods - Encouraging calming behaviors/quiet times |
| Morning routine (kids) | - Ensuring children are up and getting ready for the day (dressed in appropriate, seasonal clothes, hair/teeth brushed, bathroom needs met) | - Applying sunscreen - Taking vitamins/medicines |
| Pets | - Researching pet type/breed - Finding breeder and or rescue/shelter, researching with breeder/shelter and communication - Animal proofing if needed - Researching and interviewing veterinarian - Arranging for receiving pet - Handling vet checkup appointments (e.g. necessary shots, spayed/neutered, nails, clipping, health concerns, medicine) - Handling vet sick appointments - Determining if it makes sense to purchase pet insurance and researching insurance plans - Finding a groomer and scheduling grooming appointments Securing care during out-of-town travel/away times including researching options/ interviewing person or viewing facility - Hair pick up issues (researching vacuum options etc.) - Arranging for special outside access (e.g. dog door) - Researching and purchasing appropriate food | - Receiving pet - Purchasing food and treats and other necessary items (e.g. grooming items, bedding, crate, harness, litter) - Training (bathroom, sleeping, biting and introduction to babies/children) - Feeding: Providing food and water daily and keeping bowls clean - Outdoor access and walking: Conducting appropriate amount of walks and exercise if applicable and/or scheduling dog walker/day care - Pick up poop/empty litter box daily and weekly maintenance - Bathing and brushing and/or attending grooming appointments - Cleaning up after pet |
| Special needs & mental health (kids) | - Identifying and evaluating child’s current emotional state/special needs - Doctor/professional appointments for diagnosis and follow up - Researching and interviewing specialists for treatment such as therapists, OT, PT, speech - Discussing issues/treatment plan with child's teacher - Scheduling specialist appointments | - Working closely with “health insurance” cardholder to get coverage and medications for the issue - Advocating and meeting with school educators to secure necessary supports (i.e., IEP) - Supporting child - Attending specialist appointments |
| Discipline & screen time (kids) | - Researching appropriate discipline for different life stages - Devising a strategy to recommend to your partner - Setting up parental controls, negotiating what games/sites your kid can visit, downloading one of those apps that turns the Wi-Fi off on your child’s device after a certain number of minutes - Informing childcare helpers of limits as needed - Updating plan as needed | - Informing child and enforcing limits |
| Middle-of-the-night comfort (kids) | - Researching, creating a plan, gathering input from partner about how you want to handle your child's sleep (sleep training or a different approach) | - Executing plan for your child's sleep - Making yourself available for your children following bedtime routine, including: - Trouble falling asleep - Middle of the night feedings - Night terrors - Bed-wetting |
| Watching (kids) | - Knowing when your child will need to be watched/supervised | - Watching your child with full attention |

*Supplemental Table 3. Regression models for overall household labor*

|  |  | Depression | | |  | |  | Stress | | |  |  | Burnout | | |  | |  | Mental Health | | |  |  | | Relationship Functioning | | |  |
| --- | --- | --- | --- | --- | --- | --- | --- | --- | --- | --- | --- | --- | --- | --- | --- | --- | --- | --- | --- | --- | --- | --- | --- | --- | --- | --- | --- | --- |
| Characteristic | Beta | | 95% CI*^1^* | p-value | | Beta | | | 95% CI*^1^* | p-value | | Beta | | 95% CI*^1^* | p-value | | Beta | | | 95% CI*^1^* | p-value | | | Beta | | 95% CI*^1^* | p-value | |
| Overall household labor | .80 | | (-.62, 2.2) | .27 | | 1.5 | | | (0.23, 2.8) | .021 | | 2.8 | | (-.27, 5.9) | .073 | | -.09 | | | (-.24, .06) | .24 | | | -1.9 | | (-2.7, -1.1) | <.001 | |
| Income | -.39 | | (-1.1, .28) | .25 | | -.46 | | | (-1.0, 0.13) | .13 | | .02 | | (-1.4, 1.5) | .97 | | .01 | | | (-.06, .08) | .81 | | | -.22 | | (-.59, .16) | .25 | |
| Ethnicity | -.80 | | (-3.4, 1.8) | .55 | | -.06 | | | (-2.4, 2.2) | .96 | | -1.4 | | (-7.0, 4.3) | .64 | | .10 | | | (-.17, .37) | .47 | | | .47 | | (-.97, 1.9) | .52 | |
| Education | -1.0 | | (-1.9, -.13) | .026 | | .10 | | | (-0.69, 0.89) | .8 | | -.25 | | (-2.2, 1.7) | .80 | | .02 | | | (-.07, .12) | .6 | | | .39 | | (-.10, .89) | .12 | |
| R^2^ | 0.051 | | | | | 0.028 | | | | | | 0.013 | | | | | 0.01 | | | | | | | 0.083 | | | | |
| Adjusted R^2^ | 0.037 | | | | | 0.015 | | | | | | -0.001 | | | | | -0.004 | | | | | | | 0.07 | | | | |
| *^1^*CI = Confidence Interval | | | | | | | | | | | | | | | | | | | | | | | | | | | | |
